# Strabismus surgery in topical anaesthesia with intraoperative suture adjustment in Graves' orbitopathy

**DOI:** 10.1111/aos.16784

**Published:** 2024-10-23

**Authors:** Olav H. Haugen, Anne Elisabeth Christensen Mellgren, Maren Norli, Hans Olav Ueland

**Affiliations:** ^1^ Department of Ophthalmology Haukeland University Hospital Bergen Norway; ^2^ Department of Clinical Medicine K1, Faculty of Medicine University of Bergen Bergen Norway

**Keywords:** adjustable sutures, Graves' orbitopathy, strabismus, topical anaesthesia

## Abstract

**Purpose:**

To report the results of strabismus surgery in a series of patients with Graves' orbitopathy (GO), using topical anaesthesia with intraoperative suture adjustment.

**Methods:**

All first‐time strabismus surgeries in patients with GO in our department during the years 2014–2021 (*n* = 45) were assessed retrospectively. Among these, 31% came from outside our health region due to increased complexity of the strabismus condition. Orbital decompression surgery had been carried out in 58% of the patients prior to strabismus surgery. Patients with less than 2 months of follow‐up were excluded from the study. Median follow‐up time was 22 months.

**Results:**

Among the total patient material, 37 (82%) could be operated with topical anaesthesia with intraoperative suture adjustment. There were no cases with triggering of the oculo‐cardiac reflex during the procedure. Among the 36 patients operated with topical anaesthesia and follow‐up time ≥2 months, 11 (31%) needed further surgery. Late overcorrection after recession of the inferior rectus was seen in 19%. At the last control examination, 32 (89%) were diplopia‐free in primary and down‐gaze position, either with or without weak prisms.

**Conclusion:**

Strabismus surgery in topical anaesthesia with intraoperative suture adjustment appears to be a suitable and safe procedure in most patients with GO, including difficult and complex cases. The patients should be informed about the possibility of additional surgery and/or post‐operative need for prism glasses.

## 1 INTRODUCTION

Graves' disease is an autoimmune disorder affecting the thyroid gland, which in most cases causes hyperthyroidism. The incidence in Northern Europe is reported to be 21/100.000, with a four‐fold higher incidence in females compared to males (Abraham‐Nordling et al. 2011). Approximately, one in five patients with Graves' disease develops Graves' orbitopathy (GO), with involvement of orbital tissue, including the extraocular muscles. This often leads to disfiguring proptosis and diplopia (Shan & Douglas 2014). GO has an active first phase, characterized by inflammation of the orbital tissues, leading to swelling and exophthalmos. In the second phase, the inflammation subsides, followed by proliferation of connective tissue, leading to fibrosis of the extraocular muscles. This changes the properties of the extraocular muscles profoundly, with loss of elasticity, eventually leading to shortening/scarring. This in turn causes misalignment and severe restriction of ocular motility, which manifests in the patient as diplopia.

In case of disfiguring exophthalmos, orbital decompression may be indicated. If the exophthalmos is moderate, removal of the lateral wall is most often the chosen procedure, as this surgery seldom affects the ocular alignment. However, in some patients, the orbitopathy may be sight‐threatening, either due to compression of the optic nerve, or because the severe exophthalmos leads to exposure keratopathy, corneal ulceration and keratitis. In these cases, more radical surgery is necessary in order to expand the orbital space maximally, often by removing the infero‐medial orbital wall. However, medial orbital wall decompression changes the orbital anatomy considerably, which nearly always causes a further worsening of the eye alignment (Zloto et al. 2017).

Apart from the disfiguring and sometimes vision‐threatening exophthalmos, the misalignment and eye motility restriction are the most challenging parts of the GO. Whereas decompression surgery causes the greatest improvement in appearance scores in quality of life (QoL)‐studies, strabismus surgery and the elimination of diplopia yield the highest scores on visual functioning (Woo et al. 2022).

Due to the loss of normal elasticity of the extraocular muscles in GO, the normal dose–response relationship for strabismus surgery is fundamentally changed, and the usual dosage tables for strabismus surgery are not appropriate. However, some authors have proposed specific dosage‐response tables for strabismus surgery in GO patients (Lyu et al. 2016, Akbari et al., 2020). These patients are usually operated in general anaesthesia, with recessions on adjustable sutures according to a pre‐operatively planned dosage, and then adjusted a couple of hours after surgery (Pratt‐Johnson & Tillson 2001). Others have reported good results by reattaching the muscle at the intraoperative relaxed position, and not using adjustable sutures (Sarici et al. 2018).

In 1981, Boergen (1981) first reported the use of topical anaesthesia with intraoperative suture adjustment in this patient group. Since this first paper, he and his group have published several papers on this technique (Boergen 1993, Kalpadakis et al. 2002, Kalpadakis et al. 2004). We have used this technique in most GO patients at Haukeland University Hospital for more than 20 years. The aim of this study was to evaluate the results of strabismus surgery in these patients. Our main outcomes were intraoperative complications, frequency of reoperations, and the presence/absence of post‐operative diplopia in the primary position and down‐gaze.

## 2 MATERIALS AND METHODS

### 2.1 Patients

We have retrospectively examined the medical records of all patients with GO who underwent first‐time strabismus surgery at our department during the years 2014–2021. In total, 45 patients were included. Haukeland University Hospital is the main hospital for the Western health region of Norway (approximately 1.1 million inhabitants), primarily serving the Western part of the country. However, for many years, Graves' disease has been a field of special interest and clinical research in our department. Hence, due to the complexity of the strabismus in GO, many patients are referred to our department also from other health regions of Norway. During the study period, 14 patients (31%) came from outside our health region. In 26 patients (58%), orbital decompression had been performed prior to strabismus surgery.

**FIGURE 1:**
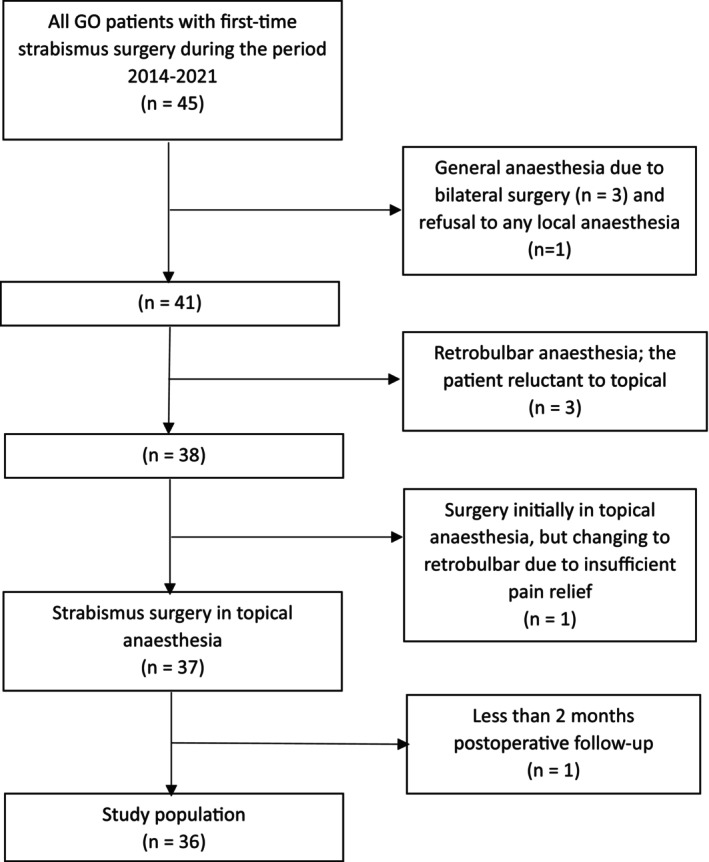
Flow chart of the included GO patients in the present study.

Due to large deviations and the need for bilateral surgery, general anaesthesia was chosen for three patients. One additional patient insisted on general anaesthesia, while another three were operated on retrobulbar anaesthesia as they were reluctant to the topical anaesthesia procedure. In one case, the surgery started in topical anaesthesia but had to be converted to retrobulbar anaesthesia due to insufficient pain relief. Thus, 37 patients (82%) of all the referred Graves' strabismus patients underwent surgery in topical anaesthesia. One of these patients had less than 2 months of post‐operative follow‐up and was therefore excluded from the study. The other 36 patients comprise the final study population (Figure 1).

### 2.2 Clinical data

The following data were extracted from the medical records: gender, age of onset for Graves' disease, age of onset for eye symptoms (GO), any decompression surgery, direction of strabismus, pre‐operative angle of deviation, pre‐operative diplopia, age at first strabismus surgery, surgical procedure, which eye and which muscle operated, intraoperative complications, post‐operative angle of deviation and presence or absence of diplopia at the routine post‐operative control examination, presence or absence of diplopia at the last control examination, the need of additional surgery and follow‐up time (from surgery to last control examination).

### 2.3 Surgical routines and technique

The following describes our routines when performing strabismus surgery in Graves' patients. At the pre‐operative examination, the patient is given thorough information about the procedure and the importance of their cooperation. In addition, the patient is also informed that post‐operative use of prisms may be necessary, as well as the possibility of further surgery.

On the day of surgery, prior to the surgical procedure, the patients are offered 15 mg of oxazepam, and an intravenous catheter is placed on the forearm. The technique of strabismus surgery in topical anaesthesia with intraoperative adjustment is carried out with the patient lying down at the operating table. Both eyes are uncovered, and oxybuprocain drops are given shortly before starting the operation, and repeated if necessary, during surgery. The patients are always monitored with electrocardiography during surgery, and atropine for intravenous injection is easily available in the operating room, due to the possibility of triggering of the oculo‐cardiac reflex.

The conjunctiva is opened with a standard limbal approach, and the selected extraocular muscle is exposed with a muscle hook. A double‐armed Vicryl 6–0 suture is placed just behind the muscle insertion, the muscle is detached and a preliminary recession with a hang‐back technique is performed. A cover test is then carried out while the patient is encouraged to fixate a light source in the primary position. If still a fixational movement, we readjust the suture and repeat the cover test. We also take care to do the same examination in downgaze. When doing recessions on the inferior rectus, special care is observed for the downgaze position in order not to overcorrect. In these cases, we accept some hypotropia in the primary position. When no or little fixational movement, the knot is permanently tied. The conjunctiva is closed with Vicryl 7‐0 Rapid and the eye is covered with chloramphenicol ointment and an eye dressing.

### 2.4 Post‐operative follow‐up

The patients are shortly examined the day after surgery, and a 7‐day prescription of antibiotic eye drops (or eventually combined with steroids) is given. The results are evaluated about 3 months post‐operatively. Due to the complexity of the strabismus in GO, as well as the need for additional lid surgery, many of the patients in the present study have been followed for a longer time, either at the outpatient clinic in our department or at other eye departments, after the strabismus surgery. Thus, in addition to the post‐operative results of the 3 months' examination, we have also recorded the results of the orthoptic examination at the last visit, whether at our department or at the local eye department. Median follow‐up time in the present study was 22 months (range 2–74).

### 2.5 Ethics

The study was approved by the Regional Committee for Medical and Health Research Ethics, Western Norway, as a quality improvement study (ref. 491 136).

### 2.6 Statistical analyses

The data were analysed using the Statistical Package for the Social Sciences (SPSS Version 26.0; IBM Corporation, Armonk, NY, USA). Continuous parametric data were reported as median (range).

## 3 RESULTS

### 3.1 Patient characteristics

Pre‐operative clinical characteristics of the patients in the study group are given in Table 1. As shown, most of the patients had hypotropia (median pre‐operative angle of deviation 27.5 prism dioptres, range 8–45), esotropia (median pre‐operative angle of deviation 27.5 prism dioptres, range 10–50), or a combination of these misalignments. Among the six patients with combined horizontal and vertical strabismus, the vertical component was the most prominent part in five. All patients had pre‐operatively diplopia; however, one of the patients could work with single vision due to strong vertical and horizontal prism glasses.

**TABLE 1:** Clinical characteristics of the study group (*n* = 36).

Variable	Results
Median age of onset, Graves' disease, years (range)	51.0 (26–76)
Median age of onset, eye manifestations, years (range)	58.0 (30–78)
Median age at first strabismus surgery, years (range)	61.0 (33–78)
Previous decompression surgery, *n* (%)	
Bilateral medial decompression	8/36 (22.2)
Unilateral lateral decompression	5/36 (13.9)
Bilateral lateral decompression	3/36 (8.3)
Medial and lateral decompression (balanced)	3/36 (8.3)
Unilateral medial decompression	1/36 (2.8)
Strabismus type, *n* (%)	
Hypotropia	16/36 (44.4)
Esotropia	12/36 (33.0)
Combined hypo and esotropia	5/36 (13.9)
Hypertropia	2/36 (5.6)
Combined hypo and exotropia	1/36 (2.8)
Diplopia, *n* (%)	36 (100)

### 3.2 Surgical procedure

All first‐time operations were unilateral recessions, on the inferior rectus (*n* = 21), the medial rectus (*n* = 13) or the superior rectus (*n* = 2), respectively. All surgical procedures were carried out by an experienced strabismus surgeon (OHH or AECM). There were no intraoperative complications, in particular, no triggering of the oculo‐cardiac reflex. On the first post‐operative day, four patients had corneal erosion, probably due to a loose eye dressing. They all healed on standard treatment with antibiotic ointment. In one patient, the suture knot (surgery on the inferior rectus) eroded through the conjunctiva and had to be trimmed a couple of weeks after the initial surgery.

### 3.3 Post‐operative alignment and diplopia

**FIGURE 2:**
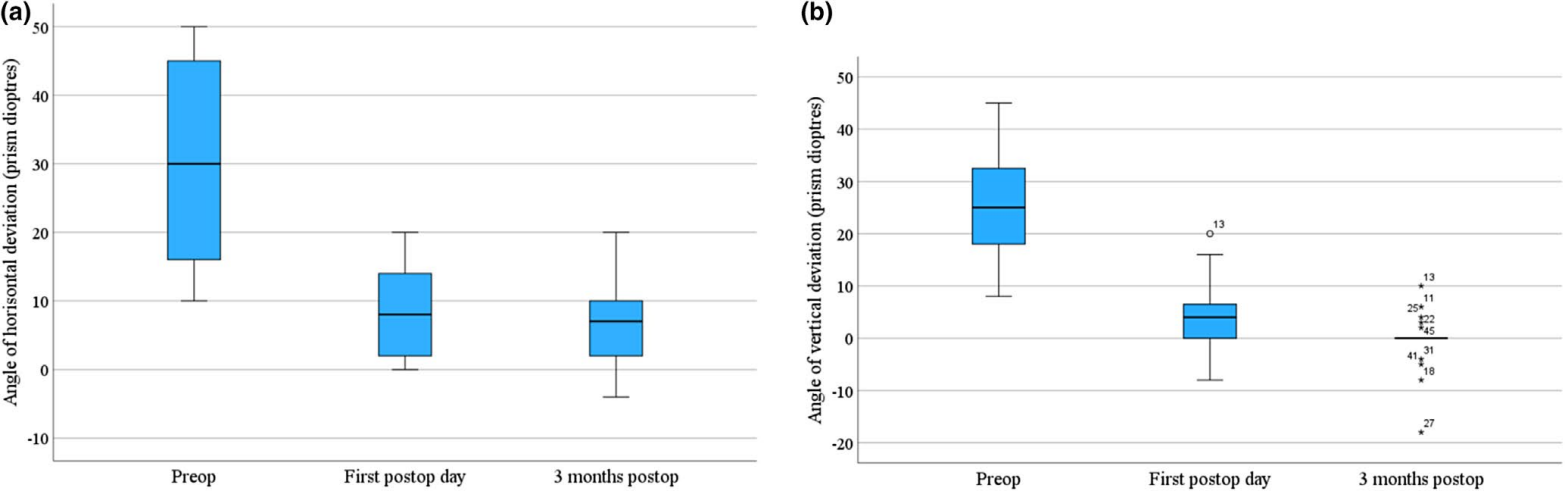
Strabismus angle of deviation pre‐operatively, on the first post‐operative day, and 3 months post‐operatively. (a) Horizontal deviation among the 13 patients with predominantly esodeviation; (b) vertical deviation among the 23 patients with predominantly vertical deviation.

Binocular single vision and a substantial improvement of the ocular alignment were present in many cases already on the first post‐operative day (Figure 2).

At the 3‐month post‐operative examination, 16 (44.4%) had binocular single vision in primary position and down‐gaze without prisms. In addition, nine (25.0%) had binocular single vision with a small prism correction. Eleven (30.6%) still had diplopia and needed further surgery. However, two of these were originally planned as a two‐step surgery due to misalignment both in the horizontal and the vertical plane, and they should not be registered as reoperations. Among the other nine patients with diplopia at the post‐operative control examination, four had undercorrections and four overcorrections, while one got a marked increase of hypotropia after surgery for esotropia (Table 2). All the overcorrections were found after recessions of the inferior rectus.

**TABLE 2:** Clinical follow‐up data on nine patients with diplopia at the 3 months' post‐operative control.

Patient ID	Initial eye muscle operated	Reason for reoperation	No. of re‐operations	Result at last control exam	Prisms needed at last control
4	IR	Overcorrection	1	No diplopia in PP and down	No
5	MR	Undercorrection	1	No diplopia for near, slight eso for distance	Yes
13	SR	Undercorrection	3	Still diplopia	Yes
14	MR	Undercorrection	0	Planned reoperation, but died	‐
18	IR	Overcorrection	1	No diplopia in PP and down	No
26	MR	Increase of hypo	1	No diplopia in PP and down	No
27	IR	Overcorrection	3	No diplopia in PP and down	Yes
41	IR	Overcorrection	2	No diplopia in PP, slight in down	No
43	MR	Undercorrection	1	No diplopia in PP and down	Yes

### 3.4 Additional surgeries

Both patients who were operated on as a planned two‐step surgery underwent a successful single second surgery and were diplopia‐free in the primary position and down‐gaze at the last control examination. Concerning the other nine patients with persistent diplopia at the 3 months' post‐operative control, the details of their individual treatment and follow‐up are presented in Table 2.

Among the 25 patients who were diplopia‐free at the 3‐months' control, either without or with prisms, there were three patients (pt# 22, 32 and 36) who experienced a re‐occurrence of strabismus/diplopia a long time (10–56 months) after the initial strabismus surgery. In one of these cases (#22), this was due to a marked worsening of exophthalmos, making a bilateral medial decompression necessary (no initial decompression), which in turn caused a large esotropia and right hypotropia. This patient needed four additional strabismus surgeries, all in topical anaesthesia, which eventually made her free of diplopia. The two other cases were esotropias that after long‐term stability gradually increased, making additional surgery necessary, after which they again became diplopia‐free without prisms. Clinical details at the 3‐months post‐operative examination, as well as the need for reoperations, are given separately for the patients with predominantly vertical and horizontal deviations in Tables 3 and 4, respectively.

**TABLE 3:** Post‐operative results (3 months) for 23 GO patients initially operated for vertical strabismus (pure or predominantly hypo‐ or hypertropias with either eso‐ or exo‐components).

ID	Strab type	Pre‐operative dev, vertic (Δ)	Pre‐operative dev, horis (Δ)	Post‐operative dev, vertic (Δ)	Post‐operative dev, horis (Δ)	Number of reop	Post‐operative control exam	Comments
2	Hypo	+30	0	0	0	0	SV	
4	Hypo	+16	0	0	0	1	D	Small overcorrection in downgaze
8	Hypo	−45	0	0	0	0	SV (P)	
11	Hypo	−23	0	0	+6	0	SV (P)	
18	Hypo	−45	0	+8	−14	1	D	Small overcorrection
20	Hypo	+16	0	0	0	0	SV	
22	Hypo	+45	0	+2	0	4	SV	Late recurrence^a^
25	Hypo	+30	0	+4	0	0	SV (P)	
28	Hypo	−30	0	0	0	0	SV	
30	Hypo	−10	0	0	−2	0	SV	
33	Hypo	−18	0	0	+2	0	SV	
37	Hypo	−35	0	0	0	0	SV	
40	Hypo	+25	0	0	0	0	SV	
41	Hypo	−18	0	+5	0	1	D	Small overcorrection
42	Hypo	+35	+7	0	0	0	SV	
45	Hypo	+16	+3	+3	−4	0	SV (P)	
13	Hyper	−35	0	−10	0	3	D	Undercorrection
21	Hyper	+30	0	0	0	0	SV	
16	Hypo + eso	−25	+10	0	+10	0	SV	Gradual decrease of esotropia
23	Hypo + eso	−25	+18	0	+45	1	D	Planned reop for esotropia
27	Hypo + eso	−22	+14	+18	+9	3	D	Overcorrection
31	Hypo + eso	+8	+7	−4	+8	0	SV (P)	Small overcorrection
35	Hypo + exo	−30	−16	0	0	0	SV	

*Note*: Horisontal: minus (−) = exodeviation, plus (+) = esodeviation. Vertical: minus (−) = left‐over‐right; plus (+) = right‐over‐left.

Abbreviations: D, double vision, also with prisms; SV, single vision without prisms; SV (P), single vision with prisms.

^a^ Initially good result, single vision without prisms. Three years later reactivation of Graves' orbitopathy, operated with medial decompression, resulting in a large esodeviation. Additional 4 strabismus surgeries later (all in topical anaesthesia), straight eyes without diplopia.

**TABLE 4:** Post‐operative (3 months) results for 13 GO patients initially operated for horizontal strabismus (pure or predominantly esotropia with a vertical component).

ID	Strab type	Pre‐operative dev, vertic (Δ)	Pre‐operative dev, horis (Δ)	Post‐operative dev, vertic (Δ)	Post‐operative dev, horis (Δ)	Number of reop	Post‐operative control exam	Comment
5	Eso	0	+30	0	+20	1	D	Undercorrection
12	Eso	0	+22	0	+8	0	SV (P)	SV (P) at 3 months; after 1 year of diplopia not responding to prisms
14	Eso	0	+45	0	+14	0	D	Undercorrection; reop planned, but the patient died
15	Eso	0	+10	0	2	0	SV	
17	Eso	0	+14	0	−4	0	SV (P)	
19	Eso	0	+16	0	0	0	SV	
26	Eso	−2	+25	−12	+10	1	D	Reop for hypotropia
32	Eso	0	+30	0	+4	1	SV	Late recurrence^a^
34	Eso	0	+14	−3	0	0	SV (P)	
36	Eso	0	+40	+2	+7	1	SV (P)	Late recurrence^b^
43	Eso	+8	+45	0	+14	1	D	Undercorrection
44	Eso	0	+50	0	+5	0	SV	
39	Eso + hypo	−18	+45	−16	+10	1	D	Planned reop for hypotropia

*Note*: Horisontal: minus (−) = exodeviation; plus (+) = esodeviation. Vertical: minus (−) = left‐over‐right; plus (+) = right‐over‐left.

Abbreviations: D, double vision, also with prisms; SV, single vision without prisms; SV (P), single vision with prisms.

^a^ Initially good result, single vision without prisms. Ten months after primary surgery increase in esodeviation and diplopia; reoperation with resection of lateral rectus, good result and no diplopia.

^b^ At the 3 months' post‐operative control a small undercorrection, but single vision with prisms. Deterioration after 4 years, reop with good result.

At the last follow‐up examination, 32/36 (88.9%) were diplopia‐free, either without (19/36 or 52.8%) or with weak prisms (13/36 or 36.1%).

## 4 DISCUSSION

In the present study, 82% of the patients with GO referred to our department with strabismus could be operated with topical anaesthesia with intraoperative suture adjustment. As many of the GO patients are of senior age, it is a great advantage to avoid general anaesthesia. There were no intraoperative complications, especially no triggering of the oculo‐cardiac reflex.

As demonstrated in Figure 2, a gradual increase in the effect of surgery was seen from the first post‐operative day to the 3‐month control examination. This was especially seen in the vertical strabismus group, as all the four overcorrections were found in this group (Table 2 and Figure 2). These patients underwent reoperations with the advancement of the inferior rectus, and in two cases it had to be repeated. Overcorrections after recession on the inferior rectus in GO strabismus patients, especially when using adjustable sutures, is a well‐known problem. The frequency of such overcorrections varies from 20% to 50% (Sprunger & Helveston 1993, Cormack et al. 2007, Volpe et al. 2012, Barker et al. 2017). In the majority of these studies, post‐operative adjustment of the sutures is performed on the same day or the day after surgery. We find it encouraging that using intraoperative suture adjustment resulted in a slightly lower rate of inferior rectus overcorrections (4/21, 19%).

We acknowledge that all Graves' patients are not suitable for strabismus surgery in topical anaesthesia. This may be due to a general fear of surgery on the eye while awake. In addition, in cases with extremely large deviations, either horizontally or vertically, bilateral surgery is often needed, and for these patients, we choose to operate in general anaesthesia. In our patient material, there were three such patients.

Previous experience with topical anaesthesia in GO strabismus patients has led us to only operate on one extraocular muscle in one sitting. In the present patient material, most of the cases had either (or predominantly) a horizontal or a vertical deviation. For instance, some of the esotropia patients had a smaller hypotropia component and vice versa. In these cases, we always operate (recessions) on the predominant deviation. In many cases, this surgical procedure also diminishes or even straightens the smaller combined deviation. However, as pointed out above, post‐operative prisms or even additional surgery may be necessary, and it is important to inform the patients about this possibility prior to surgery.

In cases with equally or near equally large deviations both horizontally and vertically, one should plan for a two‐step surgery, which should be explained to the patient in the beginning. In our material, we had two patients in this category.

In spite of a selection of the patient material towards more complicated cases, it is encouraging to conclude that almost 90% of our strabismus cases with GO operated with topical anaesthesia and intraoperative suture adjustment were diplopia‐free in primary position or down‐gaze at the last control examination, either with or without weak prism corrections.

Boulakh et al. (2022) have recently reported on strabismus surgery in GO using topical anaesthesia. Their surgical technique is somewhat different from ours, as they perform a preliminary reattachment 4 mm behind the insertion and a bow‐tie adjustable suture through the original insertion, with adjustment shortly after the surgical procedure. They reported a success (absence of diplopia in primary position and down‐gaze without prisms) rate of 95%, which is substantially better than in our study. However, they had a shorter follow‐up time (only 6 weeks), and there was no information about previous orbital decompression surgeries. In our patient material, 56% had been operated on with orbital decompression surgery, in most cases with bilateral medial decompression, which is known to seriously aggravate the ocular misalignment (Gulati et al. 2015). In addition, more than 30% of the patients were referred from other regions of the country due to high complexity of the strabismus condition. Taken together, our patient material had clearly a substantial selection towards more difficult cases.

In GO, there is no uniform consensus on success criteria after strabismus surgery. As diplopia is one of the most bothersome complaints in GO, several authors have defined the absence of diplopia as the main success criterium, especially in the important primary and downgaze/reading positions, or generally improvement of the field of binocular single vision (Gilbert et al. 2005, Nassar et al. 2009). Others have argued for more strict criteria including the use of a quality‐of‐life questionnaire (Jellema et al. 2015). Thus, the success rate varies substantially in different studies. We believe that thorough and honest information to the patient before surgery is of particular importance in these difficult strabismus conditions, in order to give the patient realistic expectations concerning the post‐operative results.

As our study has a retrospective design, it has some weaknesses and limitations. We did not record quantitatively the pre‐ and post‐operative field of single binocular vision on Goldmann perimeter, as suggested by Jellema et al. (2015); nor did we do any quality‐of‐life measurements. Concerning topical anaesthesia, we did not perform any formal scoring of the patient's experience with this procedure, as Boulakh et al., 2022 have done. Our impression, however, through observing and communicating with the patient during the surgical procedure and at the control examination on the first post‐operative day, is that the procedure in nearly all cases was well tolerated.

## 5 CONCLUSION

The strabismus in GO is one of the most challenging conditions of ocular misalignment, as the ocular motility often is severely restricted, especially after orbital decompression surgery. Surgery in topical anaesthesia with intraoperative adjustment seems to be a safe, suitable, and well‐tolerated procedure, also in complicated cases, as in this series. With this technique the risk of general anaesthesia in older and often multi‐morbid patients can also be avoided. Prior to surgery, the patients should be carefully informed about the possibility of reoperations and post‐operative need for prism glasses.

## CONFLICT OF INTEREST STATEMENT

None of the authors have any conflicts of interest.
